# Progress in the Study of Chemical Structure and Pharmacological Effects of Total Paeony Glycosides Isolated from *Radix Paeoniae Rubra*

**DOI:** 10.3390/cimb46090601

**Published:** 2024-09-12

**Authors:** Yumu Sun, Taiyu Liu, Xueying Zhao

**Affiliations:** School of Basic Medical Sciences, Heilongjiang University of Chinese Medicine, 24 Heping Road, Harbin 150040, China; 17703607675@163.com (Y.S.); 13946539517@163.com (T.L.)

**Keywords:** *Radix paeoniae rubra*, total paeony glycosides, chemical constituents, red peony, pharmacological effects

## Abstract

*Radix paeoniae rubra*, known as red peony root, is derived from the dried roots of *Paeonia lactiflora pall* or *Paeonia veitchii lynch* from the Ranunculaceae family. It is recognized for its properties of clearing heat, cooling blood, dispelling stasis, and alleviating pain, making it one of the most commonly used herbs in traditional Chinese medicine. Total paeony glycosides (TPGs) are identified as the principal active constituents of *Radix paeoniae rubra*, comprising monoterpenoid compounds with a cage-like pinane structure and monoterpenoids with a lactone structure. This review summarizes the chemical constituents and pharmacological effects of TPGs, with the aim of elucidating their relationships.

## 1. Introduction

*Paeonia lactiflora pall* (PLP) belongs to the genus Paeonia of the Ranunculaceae family, which includes 22 species and 13 subspecies. It is a perennial herb and is widely distributed across northeast and north China, southern Shaanxi, and Gansu, with its presence also noted in North Korea, Japan, Mongolia, and the Siberian region of Russia [1]. The primary medicinal varieties are *radix paeoniae rubra* (RPR) (also known as *paeoniae radix rubra*) and *Radix Paeoniae Alba* (RPA), with the medicinal part being the dried root. In the 2020 edition of the Chinese Pharmacopoeia, the botanical source of RPA is attributed to *Paeonia lactiflora pall*, while RPR is derived from either *Paeonia lactiflora pall* or *Paeonia veitchii lynch* [2]. In addition to the specified sources in the Chinese Pharmacopoeia, other varieties such as *P. obovata Maxim.*, *P. obovata* subsp. *willmottiae (Stapf) D.Y. Hong* and *K.Y. Pan*, *P. mairei H. Lév.*, *P. lactiflora* var. *trichocarpa (Bunge) Stern*, *P. emodi Royle*, *and P. sterniana H.R. Fletcher* are considered the substitutes for RPR in regions, like Xizang in China [3,4,5]. Recent studies showed that RPR contains monoterpenes, triterpenes, lignans, flavonoids, and volatile oils that have pharmacological effects on the cardiovascular, nervous, and digestive systems. In the treatment of blood stasis, its mechanism of action has been more thoroughly studied, which is an aspect that significantly distinguishes it from RPA [6]. Additionally, the differences in botanical sources, processing, and harvesting methods also distinguish RPA and RPR, with RPA defined as the boiled and peeled dried roots of PLP and RPR as the dried roots of *Paeonia lactiflora* or *Paeonia veitchii lynch*. The 2020 edition of the Chinese Pharmacopoeia highlights variations between the two in terms of flavor, meridian tropism, efficacy, and indications, as shown in Table 1 [2].

The differences between the two medicines in terms of active ingredients are determined by the differences in their basal origin, processing and harvesting, place of origin, flavors and properties, and efficacy and main treatment [7]. Feng et al. [8] extracted paeoniflorin, albiflorin, and oxypaeoniflorin from separate RPR and RPA and treated Sprague Dawley (SD) rats by gavage to collect blood for detection. Their study found that the absorption rate of paeoniflorin from RPR was higher than that from RPA, while the absorption rates of albiflorin and oxypaeoniflorin from RPR were lower than those from RPA, which showed that there were differences in the absorption and metabolism rates between the two. Total paeony glycosides (TPGs) is the term for all monoterpene glycosides extracted from RPR [6,9]. The primary monoterpene glycosides common to TPGs include paeoniflorin, albiflorin, oxypaeoniflorin, benzoyl paeoniflorin, and benzoyl hydroxy paeoniflorin [6,10]. Paeoniflorin is notably the most abundant and the best activity among them. The disparity in paeoniflorin levels serves as a distinguishing marker between RPR and RPA. Although the extraction techniques can influence paeoniflorin content, the Chinese Pharmacopoeia (2020 edition) mandates that RPA must contain no less than 1.6% paeoniflorin, while RPR must contain at least 1.8%. Some studies have demonstrated that RPR exhibits greater anti-allergic effects than RPA, primarily due to the moderate anti-allergic activity of paeoniflorin-type monoterpene glycosides compared to those of albiflorin type [11]. Jia et al. [12] conducted high-performance liquid chromatography (HPLC) analyses of RPR and RPA and found paeoniflorin levels of 41.24% in RPR versus 11.78% in RPA. Moreover, the concentration of oxypaeoniflorin was higher in TPGs, whereas albiflorin was more prevalent in TPGs. These findings [13] were corroborated by another study. Interestingly, Lin Peng et al. [14] found that 67.6% of paeoniflorin could be extracted from RPA by optimizing the extraction technique, so optimizing the extraction technique is also an important tool for studying TPGs. In addition to the differences in the content of the active ingredients, there are also differences in the pharmacokinetic characteristics of the same active ingredients.

In summary, although RPR and RPA both belong to the Peony species and share some botanical sources, they exhibit distinctions in origin, processing, flavor, meridian tropism, efficacy, indications, and active components. Moreover, traditional Chinese medicine has different descriptions of its efficacy, and there are obvious differences in the clinical application of traditional Chinese medicine. Recently, Fan et al. provided a comprehensive review of the traditional uses, phytochemistry, pharmacological activity, and therapeutic mechanism for BSS and quality control of *Paeoniae Radix Rubra* [6]. However, based on the role of TPGs as the main active ingredient in RPR, a review of the recent literature reveals a scarcity of comprehensive reviews specifically addressing TPGs. Therefore, this article aims to collate the relevant literature to summarize the chemical structures and pharmacological actions of major monoterpenoid glycosides in TPGs, providing a reference for the further development and utilization of TPGs from *Radix paeoniae rubra*.

## 2. Chemical Structure of TPGs

**Figure 1:**
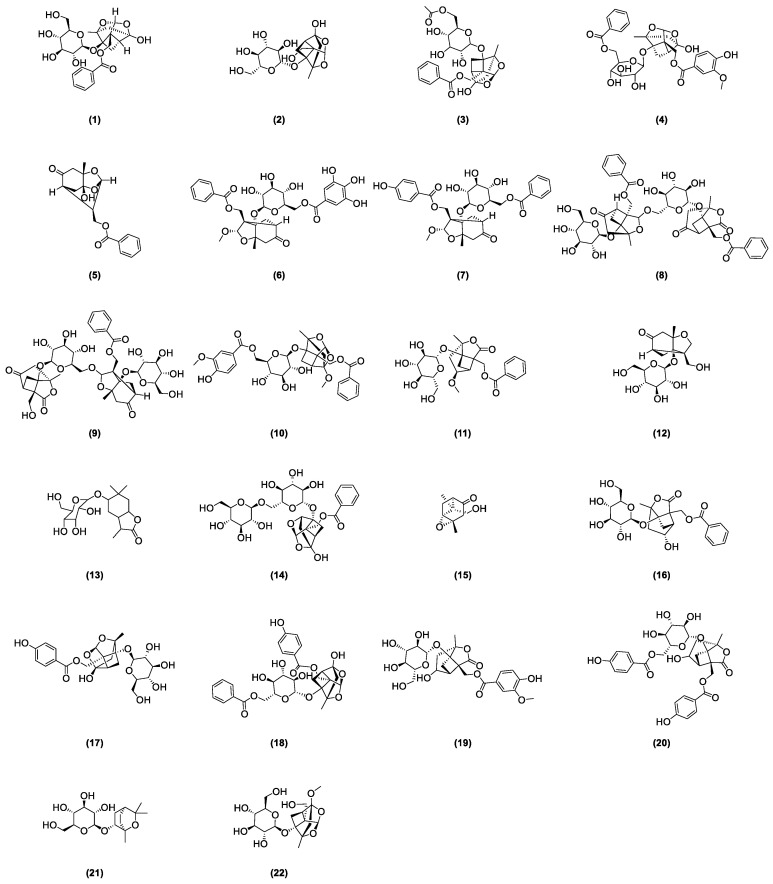
Some glycoside compounds isolated from *Radix paeoniae rubra*.

As stipulated by the “Pharmacopoeia of the People’s Republic of China”, paeoniflorin is the indicative component for quality control of *radix paeoniae rubra* [2]. This compound was first isolated from the roots of a peony by Shabata et al. in 1963 [15], yet other glycoside compounds also abound in *radix paeoniae rubra*. In recent years, many scholars developed various analysis methods to isolate and identify the chemical structures of major monoterpenoid glycosides in TPGs. Figure 1 depicts the glycoside compounds isolated from *Radix paeoniae rubra*. Hsu et al. [16] applied spectroscopic analysis techniques, such as 1H-NMR and 13C-NMR, to identify paeoniflorin (1) and 8-Debenzoylpaeoniflorin (2). Through fast atom bombardment mass spectrometry (FAB-MS) and NMR technologies, Wu and colleagues [17] extracted and analyzed acetoxypaeoniflorin (3) from RPR. Li and the team [18] analyzed the acetone extract of *Paeonia anomala* subsp. veitchii, identifying compounds such as 3″-methoxy-4″-hydroxy-6′-benzoylpaeoniflorin (4) and paeoniflorigenone (5) through UV, MS, and 1H-NMR methods. Duan [19] developed a precise, rapid, and convenient P-TLC and P-HPLC method to extract and isolate compounds like paeoniflorin B (6), C (7), F (8), G (9), 6′-*O*-vanillylpaeoniflorin (10), 4-methylalbiflorin (11), 1-*O*-*β*-D-glucopyranosyl-paeonisuffrone (12), 6-*O*-*β*-D-glucopyranosyl-lactinolide (13), 6′-*O*-*α*-glucopyranosylpaeoniflorin (14), and paeoniveitone (15). Liu et al. [20] applied HPLC, in conjunction with diode array detection (DAD) and electrospray ionization time-of-flight mass spectrometry (ESI-TOFMS), to develop a rapid, sensitive technique for analyzing and identifying compounds in RPR, successfully determining 26 components, including monoterpenoid glycosides such as albiflorin (16), oxypaeoniflorin (17), and benzoyloxy-paeoniflorin (18). Bi et al. [21] identified two new monoterpenes, 4″-hydroxy-3″-methoxy-albiflorin (19) and 6′-*O*-*p*-hydroxybenzoyl-4″-hydroxyalbiflorin (20), from the ethanol extract of RPR using 1H-NMR and 13C-NMR. Liang et al. [22] used HPLC-DAD-ESI-IT-TOF-MS to research PRR decoction and isolated two monoterpenoid glycosides: 1,8-cineole-2-*O*-*β*-d-glucopyranoside (21) and 4-*O*-Methyldesbenzoylpaeoniflorin (22).

**Figure 2:**
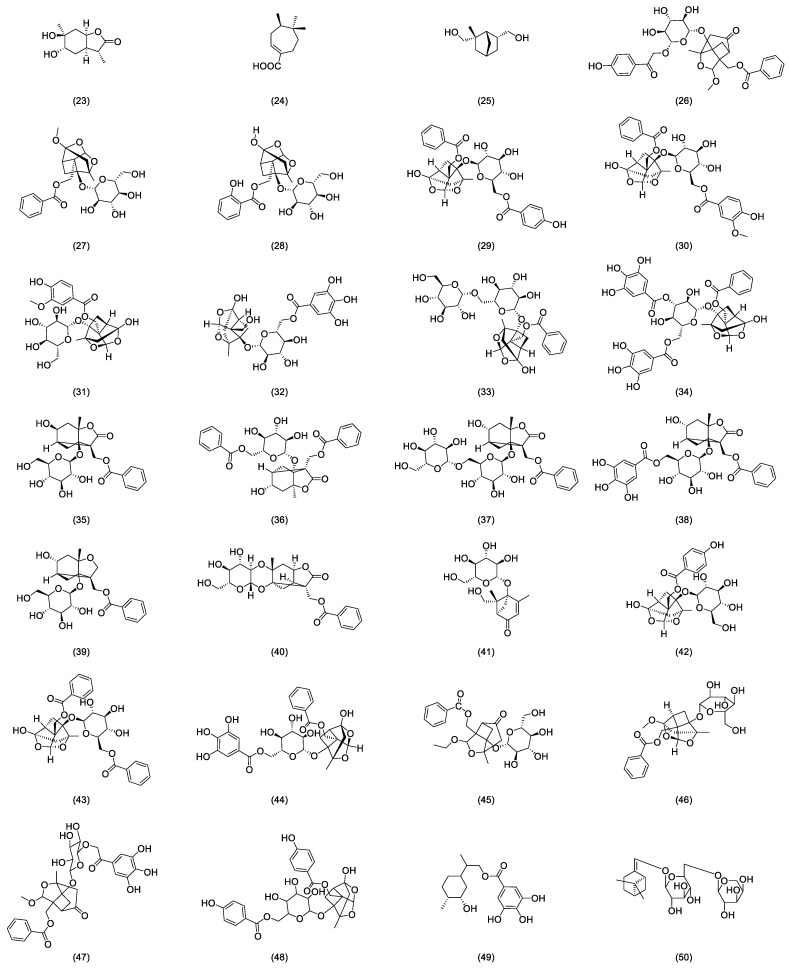
Some glycoside compounds isolated from *Radix paeoniae rubra*.

In nearly a decade of research, Liang et al. [23] extracted and isolated five compounds from the root of RPR and confirmed the presence of three monoterpenes, paeoveitols A (23), B (24), and C (25), using infrared (IR) spectroscopy, ultraviolet (UV) spectroscopy, and MS (Figure 2). Shi et al. [24] isolated paeonidanin E (26) from the roots of a peony using HPLC (Figure 2). They [25] analyzed the species of the genus paeonia and found 4-*O*-methyl-paeoniflorin (27) to be unique to Veitch’s peony, distinguishable from white a peony, and detected compounds salicylpaeoniflorin (28), mudanpioside C (29), J (30), E (31), 6′-*O*-galloyldesbenzoyl-paeoniflorin (32), isomaltopaeoniflorin (33), 3′,6′-di-*O*-galloylpaeoniflorin (34), 4-epi-albiflorin (35), paeonivayin (36), 6′-*O*-glucopyranosylalbiflorin (37), galloylalbiflorin (38), paeoniflorol (39), lactiflorin (40), and mudanpioside F (41) (Figure 2). Gu et al. [26] analyzed sliced RPR to obtain oxypaeoniflora (42) and benzoylpaeoniflorin (43) (Figure 2) by utilizing UPLC/Q-TOF-MS with electrospray ionization (ESI). Zhang et al. [27] applied HPLC-DAD-ESI-IT-TOF-MS to analyze the freeze-dried RPR powder, isolating galloylpaeoniflorin (44) (Figure 2). Jin et al. [28] established a simple, accurate, and rapid UHPLC-Q-Exactive-Orbitrap-MS method to identify compounds from RPR, including 9-ethyllactiflorin A (45), 4-methylpaeoniflorin (46), paeonidanin D (47), mudanpioside H (48), and paeonidanin H (49) (Figure 2). Furthermore, RPR is known to contain (Z)-(1S,5R)-β-Pinen-10-yl-β-vicianoside (50) [29] (Figure 2).

## 3. Pharmacology of TPGs

### 3.1. Effects of Improving Anticoagulation, Antithrombosis, and Microcirculation

The pharmacological actions of TPGs as the effective components of blood-activating and stasis-resolving agents have been confirmed through mechanisms such as inhibiting the GSK3β signaling pathway, modulating active substances in vascular endothelium, reducing blood viscosity, prolonging prothrombin time, and activating partial thromboplastin time, thereby achieving antithrombotic, anticoagulant, and microcirculatory effects.

#### 3.1.1. Effects of Anticoagulation and Antithrombosis

Deep vein thrombosis (DVT), characterized by the formation of blood clots within deep veins, remains one of the leading causes of morbidity and mortality worldwide [30]. Studies demonstrated that the main active components of RPR distribution granules, such as paeoniflorin and benzoylpaeoniflorin, can prevent DVT by inhibiting the glycogen synthase kinase 3β (GSK3β) signaling pathway and ameliorating NF-κB-driven local inflammation [31]. Research by Xie et al. [32] assessed the anticoagulant activity of RPR extracts on rats with blood stasis models induced by ice water immersion and subcutaneous injection of epinephrine using in vitro measurements of activated partial thromboplastin time (APTT), thrombin time (TT), prothrombin time (PT), and fibrinogen (FIB). The isolation and identification of the effective components of RPR confirmed paeoniflorin as a main antithrombotic constituent. The mechanism of their antithrombotic action may be related to the activation of anticoagulant activity in the bloodstream and the regulation of active substances within the vascular endothelium.

Sun et al. [33] employed an in vitro thrombosis method to observe the effects of TPGs on the length and dry/wet weight of thrombi; they also established a blood stasis model to examine the impact of TPGs on blood viscosity, hematocrit, platelet aggregation rate, and coagulation time in rats with blood stasis. Their findings indicate that TPGs possess antithrombotic properties, which are mechanistically linked to the protection of vascular endothelial cells from damage and a reduction in blood viscosity. Wang et al. [34] found that TPGs can significantly reduce platelet and red blood cell aggregation, enhance red blood cell deformability, prolong PT and APTT, reduce whole blood viscosity (at low shear), and decrease thrombus formation. Wu et al. [35] used coagulation assays to measure coagulation time, prothrombin time, and thrombin time in mice, as well as the activity of coagulation factors II, V, VII, VIII, IX, X, XI, and XII and plasma fibrinogen levels. The results showed that TPGs can significantly prolong coagulation time, prothrombin time, and thrombin time in mice; they can obviously reduce the activity of exogenous coagulation factors II and V and endogenous factor IX and increase the activity of antithrombin III in rats.

In studies on combined medication and synergistic formulation, Xu et al. [36] observed the anticoagulant effects of a combination of astragalus total saponins and TPGs (1:3) (Qi-Shao dual saponins). The results indicated that all three doses of Qi-Shao dual saponins could inhibit ADP-induced platelet aggregation, significantly reduce GMP-140 levels, and markedly lower the TXA2/PGI2 ratio, confirming the significant anticoagulant effects of Qi-Shao dual saponins. To enhance the antithrombotic effects of TPGs, other researchers [37,38] studied the optimal ratios of TPGs with effective components of Chuanxiong and panax notoginseng total saponins, providing experimental evidence for the synergistic pharmacological actions and compatibility of traditional Chinese medicine.

#### 3.1.2. Effects of Improving Microcirculation

Liu et al. [39] found that TPGs significantly improved the microcirculatory status of the body, reduced serum and plasma viscosity, inhibited adenosine diphosphate-induced (ADP-induced) platelet aggregation, and prolonged prothrombin time (PT) and activated partial thromboplastin time (APTT). Another study [40] showed that TPGs can significantly increase the red blood cell deformability index in hemorrhagic shock model rats, reduce red blood cell aggregation, and decrease blood viscosity, thereby improving hemorheology and reducing blood flow resistance. Additionally, TPGs can ameliorate the hypotension and bradycardia caused by hypovolemia in rats, thereby accelerating blood flow velocity to some extent and restoring blood volume.

In summary, TPGs can exert anticoagulant, antithrombotic, and microcirculation improvement effects through multiple pathways. Paeoniflorin is likely the main effective component of TPGs on antithrombotic action, with their primary mechanisms involving the prolongation of prothrombin and thrombin times. Furthermore, a specific ratio of TPGs in combination with astragalus and panax notoginseng total saponins can enhance antithrombotic and anticoagulant effects.

### 3.2. Effects of Protecting Cardiovascular Disease

#### 3.2.1. Effects of Protecting Attenuation of Myocardial Cell Apoptosis

Acute myocardial infarction (AMI) is one of the most common cardiovascular diseases, triggered by occlusion of the coronary arteries in animal models [41]. TPGs can protect against cell apoptosis induced by AMI, as evidenced by its ability to prevent an increase in the number of apoptotic cardiomyocytes, caspase-3 activity, and alterations in B-cell lymphoma/leukemia-2 (Bcl-2) expression, as well as a decrease in the ratio of Bcl-2 to Bax in rats with AMI [42]. The study by Mo et al. [42] demonstrated that TPGs had a protective effect on the changes in myocardial enzymes (GOT, CK-MB, α-HBDH, CK, and LDH), cytokines (TNF-α and IL-10), oxidative stress markers (SOD, MDA), and coagulation parameters (TT, PT, APTT) in a rat model of AMI induced by ligation of the left coronary artery. Moreover, this protective effect is dose dependent, indicating the active role of TPGs in modulating the aforementioned factors.

Ventricular remodeling (VR), a primary pathological factor in post-AMI heart failure, is characterized by thinning and dilation of the myocardium in the infarcted area, compensatory elongation of segments in the non-infarcted myocardium, and progressive spherical dilation of the left ventricle [43]. Zhang et al. [44] observed that a combination of total saponins from stems and leaves of *Panax quinquefolius* with TPGs significantly reduced serum levels of TNF-α, IL-1β, LA, PIIINP, and hyaluronidase (HA) in rats with AMI models, mitigated inflammatory responses in myocardial cells, interstitial edema, and necrotic degeneration of myocardial fibroblasts, significantly decreased left ventricular dimensions, and significantly improved left ventricular ejection fraction. Furthermore, it downregulated the expression of type I and III collagen. These results suggest that the qi-invigorating and blood-activating components improve VR by reducing inflammatory infiltration during myocardial fibrosis in AMI rat models, decreasing the release of damaged myocardial fibrosis factors and preventing excessive deposition of various types of collagens.

Persistent ischemia and hypoxia lead to myocardial infarction (MI), a necrotic myocardial lesion that produces oxidative stress and inflammatory responses, resulting in myocardial necrosis, apoptosis, and reduced cardiac function [45]. Studies showed that TPGs had cardioprotective effects and mitigated cardiomyocyte apoptosis in MI rats, which were associated with the inhibition of the RhoA/ROCK pathway [46]. Gao et al. [47] explored the protective effects of TPGs on cardiomyocyte damage induced by tert-butyl hydroperoxide (tBHP), finding that TPGs afforded a certain level of protection on tBHP-induced myocardial cell damage, potentially through mechanisms involving stabilization of the myocardial cell membrane, scavenging of free radicals, and inhibition of early cardiomyocyte apoptosis. Shen et al. [48] discovered that TPGs showed a protective effect on apoptosis of myocardial ischemic injury induced by isoproterenol in rats, with the medicinal mechanism likely related to the activation of the Bcl-2 gene and protein expression, the inhibition of the Bax gene and protein expression, and an increase in the Bcl-2/Bax protein ratio, thereby reducing myocardial cell apoptosis. Reperfusion following acute myocardial infarction (AMI) may cause structural damage to the heart, leading to sudden cardiac arrest, cardiac function decline, and the emergence of malignant arrhythmias, a phenomenon known as ischemia/reperfusion (I/R) injury. I/R injury can induce oxidative stress [49,50] and lead to varying degrees of cardiomyocyte apoptosis [51]. TPGs can upregulate the expression of caspase-3 precursor and Bcl-2 in H9C2 cells damaged by I/R in a dose-dependent manner and downregulate the expression of cleaved caspase-3, cleaved PARP1, and Bax, reducing ROS levels, LDH, and MDA activity. This inhibition of I/R injury-induced cardiomyocyte apoptosis and oxidative stress may be achieved through the suppression of the PI3K/Akt signaling pathway [52].

#### 3.2.2. Effects of Scavenging Free Radicals and Inhibiting Oxidative Stress

The role of oxidative stress is crucial in the development and progression of cardiovascular diseases. To investigate the antioxidative protective effects of TPGs on myocardial cell damage, Mo et al. [53] introduced isoproterenol into cultured neonatal rat cardiomyocytes to create a model of ischemic hypoxic injury. The results suggested that TPGs provided a protective effect on myocardial damage caused by isoproterenol, exhibiting a dose-dependent relationship. The mechanism of action was related to enhancing the antioxidative capacity of cells, reducing free radicals, and lipid peroxidation-induced cell membrane damage. Zhang [54] found that TPGs significantly reduced (*p* < 0.05) the activities of creatine kinase (CK) and lactate dehydrogenase (LDH) in H9c2 cardiomyocytes under a microwave radiation injury model and significantly lowered the content of reactive oxygen species (ROS). Another study [55] showed that TPGs not only improved the reduced levels of the glycolytic enzyme-pyruvate kinase M2 isoform, LDHA, and glyceraldehyde-3-phosphate dehydrogenase (GAPDH), but also resisted the decrease in H9c2 cell viability induced by tert-butyl hydroperoxide (tBHP), inhibiting the production of oxidants within the cell and mitochondria and maintaining the total and nuclear levels of Nrf2 protein in H9c2 cells. Long et al. [56] demonstrated that TPGs had a direct destructive effect on free radicals. Their results indicated that myocardial ischemic injury led to the production of oxygen free radicals and lipid peroxidation, and the cardioprotective effects of TPG treatment for myocardial ischemia were superior to those of propranolol. Further research indicated that the protective effects of TPGs on experimental myocardial ischemia induced by isoproterenol in rats might be achieved by scavenging free radicals and reducing oxidative damage caused by ischemia. It is evident that TPGs improve myocardial cell dysfunction by protecting the antioxidative capacity of damaged myocardium, inhibiting oxidative stress responses, and scavenging free radicals.

### 3.3. Effects of Anti-Tumor Activity

TPGs, as the main active components of RPR, exhibit therapeutic effects on various malignancies, including bladder cancer, ovarian cancer, liver cancer, lung cancer, and melanoma. Their anti-tumor efficacy is achieved through multiple, intersecting pathways.

#### 3.3.1. Effects of Immunomodulation and Induction of Tumor Cell Apoptosis

**Figure 3:**
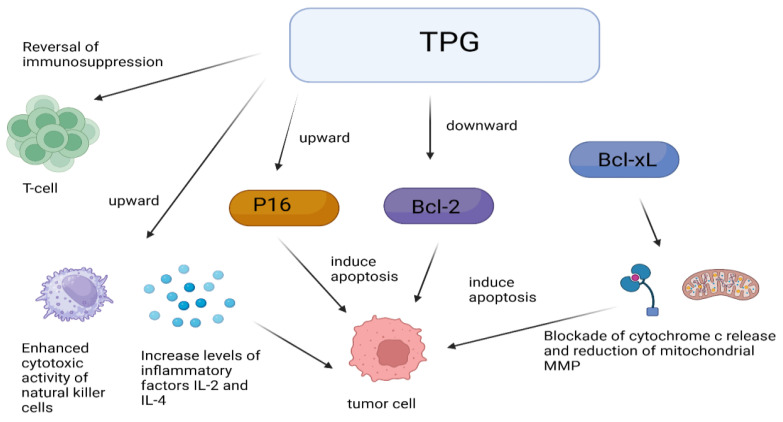
Anti-tumor mechanisms of TPGs.

One of the mechanisms of the anti-tumor effect of TPGs is through immune regulation. TPGs did not have a direct cytotoxic effect on S180 cells in vitro but can antagonize the immunosuppression induced by tumor S180 cells and cyclophosphamide (CTX), enhance the cytotoxic activity of natural killer cells, and increase the levels of inflammatory cytokines IL-2 and IL-4 to inhibit tumor cell growth, suggesting that TPGs may achieve anti-tumor effects through immunomodulation [57]. Interestingly, monoterpenes are derived from RPA, whose anti-tumor mechanisms are also associated with immunomodulation [58]. Figure 3 shows some mechanisms of anti-tumor effects by TPGs. Inducing apoptosis in tumor cells is another mechanism of the anti-tumor effect of TPGs. Fan’s experiments confirmed that within a certain range of dosing time and dosage, TPGs did not exhibit hepatotoxicity and did not affect the normal functioning of the liver, but they possess anti-tumor activity on liver cancer by inhibiting the proliferation of human hepatocellular carcinoma (HepG2) cells and inducing apoptosis [59]. Yu et al. [60] studied the regulation of immune function in tumor-bearing mice by TPGs and observed that TPGs increased the levels of interleukin-2 (IL-2) and decreased the levels of interleukin-10 (IL-10) in peripheral blood. The results suggest a dual anti-tumor action mechanism of TPGs: on one hand, reversing T lymphocyte immune suppression and correcting immune disorders; on the other hand, upregulating p16 and downregulating Bcl-2 gene proteins to induce tumor cell apoptosis. Both Bcl-2 and B-cell lymphoma extra large (Bcl-XL) can inhibit apoptosis by blocking the release of cytochrome c and reducing mitochondrial membrane potential (MMP). TPGs can inhibit the expression of Bcl-2 and Bcl-XL in K562 cells, promote the expression of Bcl2-associated X (Bax), and cause the accumulation of cytochrome c, caspase-9, and caspase-3 in the cytoplasm, thereby inducing apoptosis in K562 cells [61]. Fan et al. [62] found that TPGs can upregulate the expression of PTEN and downregulate the expression of protein kinase B (PKB, Akt) and phosphatidylin-ositol-3-kinase (PI3K), inducing apoptosis in Hep G2 cells and inhibiting their proliferation through the PTEN/PI3K/Akt signaling pathway.

#### 3.3.2. Effects of Inhibiting Tumor Cell Proliferation, Migration, and Invasion

In vitro studies demonstrated that TPGs possessed the ability to inhibit tumor cell proliferation, migration, and invasion. TPGs can exert a strong inhibitory effect on the proliferation of human hepatocellular carcinoma cells SMMC-7721 and can dose dependently suppress the migration of SMMC-7721 cells, a mechanism that may be associated with the downregulation of vascular endothelial growth factor (VEGF) protein expression and cyclooxygenase-2 mRNA expression [63]. Gao et al. [64] used the human lung cancer A549 cell model and compared it with the TPG intervention model and found that TPGs may inhibit the proliferation, migration, and invasion of human lung cancer A549 cells by suppressing the activation of the Akt pathway, thereby reducing the expression of matrix metalloproteinases-2 and -9 (MMP-9). In addition, TPGs can also downregulate the mRNA and protein expression of MMP-9 and hypoxia-inducible factor-1α (HIF-1α) in H1299 cells, exerting an anti-non-small-cell lung cancer effect [65].

#### 3.3.3. Effects of Regulating Gene and Protein Expression

TPGs achieve anti-tumor effects by regulating a variety of genes, such as tumor resistance genes and tumor suppressor genes. Research displayed that TPGs can exert therapeutic effects on lung cancer by regulating the expression of tumor resistance-related genes multidrug resistance protein (MRP), multidrug resistance1 (MDR1), and tumor suppressor genes P21, P16, and protein P53 [66]. In addition, Gu et al. [67] found that TPGs improved ciliated epithelial cell damage and alveolar sac expansion in a rat model of lung cancer, significantly reduced lung index, microvascular density, the expression of apoptosis inhibitor protein surviving, and the positive expression of the viral oncogene kirsten rat sarcoma viral oncogene. It is suggested that TPGs can effectively inhibit the expression of apoptosis inhibitor proteins and oncogenes in the lung tissue of rats with non-small-cell lung cancer, reduce the formation of microvessels, and ultimately inhibit the development of lung cancer. The secretion of angiogenic factors is one of the characteristics that distinguish tumor cells from normal cells. In the culture of melanoma A375 cells, it was found that TPGs downregulated the expression of multidrug resistance gene-1, topoisomerase II isoenzymes, multidrug resistance-associated proteins, and survivin mRNA and protein, regulated the reversal of multidrug resistance factors, and ultimately inhibited the growth of A375 cells [68].

#### 3.3.4. Effects of Cytotoxicity

Bladder cancer is one of the most common cancers among men globally, with the majority of cases histologically diagnosed as urothelial carcinoma (UC) [69,70]. Lin et al. [71] studied the therapeutic effects of the RPR extracts on bladder cancer, where the extracts primarily consisted of gallic acid and TPGs. Their research demonstrated that TPGs exhibited anti-bladder cancer activity, and intravesical therapy with these compounds reduced tumor size in mice. Moreover, TPGs exhibited cytotoxicity in bladder cancer MB49 cells of mice and showed less cytotoxicity in normal bladder SV-HUC-1 cells. Wang et al. [72] summarized multiple studies on the treatment of TPGs on various cancers, including liver cancer, stomach cancer, and glioblastoma, encompassing 13 types of cancers and 40 mechanisms of action. These mechanisms are similar to the anticancer effects of TPGs, suggesting that paeoniflorin, as the most abundant glycoside in TPGs, is likely the main active component responsible for the anticancer properties of TPGs.

In summary, the anti-tumor mechanisms of TPGs primarily involve modulating the immune system, inducing tumor cell apoptosis, inhibiting tumor proliferation and metastasis, regulating gene and protein expression, and exerting direct cytotoxic effects. Induction of tumor cell apoptosis is mainly achieved through the mitochondrial pathway, blocking the tumor cell proliferation cycle, and affecting the expression of apoptosis-related genes. Research on reversing tumor cell multidrug resistance is relatively scarce, but it undoubtedly represents one of the new pathways for anticancer therapy. Furthermore, studies on the anti-tumor effect of a single component of TPGs are few.

### 3.4. Effects of Anti-Aging

D-galactose (D-gal) is a reducing sugar that can induce non-enzymatic glycation reactions both in vivo and in vitro, leading to the formation of AGEs. The accumulation of AGEs in tissues induces oxidative stress by producing excessive ROS, which can accelerate age-related changes [73,74]. Previous studies showed that long-term injection of D-galactose induced age-related changes in mice or rats, such as neurodegenerative changes, osteoporosis, and age-related hearing loss [75,76]. Based on this theory, the D-galactose-treated mouse model has been widely used in anti-aging research. Recent studies have shown that the monoterpene glycosides in RPR have antioxidant properties that can reduce the damage caused by oxidative stress [77]. TPGs showed anti-aging pharmacological effects in three ways: inhibition of oxidative reactions, mediation of immune inflammation, and repairing brain neurons.

#### 3.4.1. Effects of Inhibiting Oxidative Reactions

Studies demonstrated that TPGs significantly reduced monoamine oxidase levels in the brain tissues of D-galactose-induced aging mice, enhanced acetylcholinesterase activity, inhibited the increase of lipid peroxidation product malondialdehyde (MDA) content, and elevated superoxide dismutase (SOD) levels in brain tissues, thereby improving learning and memory impairments in aging mice [78]. Wang et al. [79] found that TPG treatment in D-galactose-induced aging rats improved learning and memory disorders, increased brain tissue SOD activity, and reduced MDA concentration, consistent with the aforementioned studies. Additionally, the rats exhibited enhanced erythrocyte aldose reductase (AR) activity, increased plasma levels of HbA1c, fructosamine (FRA), and AGEs, and elevated tissue AGE content, suggesting that TPGs can perform anti-aging effects by attenuating glycation–oxidative stress responses, reducing lipid peroxidation products and early AGE content, and weakening AR activity. Yang et al. [80] observed the effects of TPGs on learning and memory in D-galactose-induced aging mice and related enzymes and metabolites involved in neurotransmitter regulation. The results showed that TPGs significantly improved learning and memory impairments in aging mice. In the shuttle box test, the mice exhibited a significant increase in active avoidance latency and the number of active avoidances; in the water maze test, TPGs effectively increased the correct rate of reaching the platform and reduced the number of errors. Moreover, TPGs significantly reduced monoamine oxidase levels and increased acetylcholinesterase activity in the brain tissues of aging mice; they also inhibited the increase of lipid peroxidation product MDA content and elevated the level of SOD in brain tissues. Furthermore, TPGs significantly improved immune function deficits and brain atrophy in aging mice.

#### 3.4.2. Effects of Mediating Immune Inflammation

During the aging process, the dysfunction of the immune system that leads to a chronic pro-inflammatory state is known as “inflammaging” [81]. Toll-like receptors (TLRs) [82] play a role in promoting the expression of inflammatory mediators, initiating innate immune defenses early, and activating the adaptive immune system. Zhang et al. [83] found that TPGs could improve learning and memory abilities in D-galactose-induced aging rats by regulating the expression of TLR4 mRNA and IL-33 mRNA, thus improving the condition of immune organs and atrophied brain tissues, accelerating the clearance of free radicals and inhibiting the release of inflammatory factors. These results indicate that the anti-aging effects of TPGs are also related to the mediation of immune inflammation.

#### 3.4.3. Effects of Repairing Brain Neurons

Repairing brain neurons to improve learning and memory capabilities may also be an anti-aging effects mechanism of TPGs. He et al. [84] used primary rat cortical neurons to prepare models of calcium overload injury induced by caffeine (Caf), KCl, and NMDA. The results showed that TPGs had a significant protective effect on neurons in all three calcium overload injury models, significantly increasing the survival rate of injured neurons and reducing the levels of lactate dehydrogenase (LDH) released by cells. This indicates that TPGs have a significant protective effect on calcium overload-induced neuronal injury in rats.

### 3.5. Effects of Anti-Cerebrovascular Disease

TPGs in the treatment of cerebrovascular diseases mainly include three aspects: the treatment of hypertension, the treatment of acute cerebral ischemia and ischemia/reperfusion injury, and the treatment of focal cerebral ischemia. Table 2 lists the mechanisms of TPGs in treating cerebrovascular diseases.

#### 3.5.1. Effects of Treating Hypertension

Hypertension is a primary factor in inducing cerebrovascular diseases and is closely associated with platelet activation and inflammatory responses. In hypertensive patients, mean platelet volume (MPV) is positively correlated with serum levels of high-sensitivity C-reactive protein (hs-CRP) [85]. Research by Xiao et al. [86] demonstrated that TPGs could reduce MPV and decrease serum levels of hs-CRP and soluble CD40 ligands (sCD40L) in spontaneously hypertensive rats (SHRs) as well as regulate the balance of serum nitric oxide (NO) and endothelin (ET). Therefore, these findings suggest that TPGs may treat hypertension by inhibiting platelet activation, reducing inflammatory cytokine levels, and increasing serum NO levels. As is well known, chronic hypertension leads to vascular remodeling. Studies exihibited that hypertensive vascular remodeling involved vascular oxidative stress and elevated levels of matrix metalloproteinase-9 (MMP-9) [87]. TPGs can inhibit arterial oxidative stress and reduce MMP-9 levels, thereby improving hypertensive vascular remodeling [88] (Table 2). Large fluctuations in blood pressure in long-term hypertensive patients can lead to vascular rupture and cerebral hemorrhage. Following cerebral hemorrhage, activation of microglia results in the production of a large number of inflammatory mediators, further damaging brain tissue, disrupting the blood–brain barrier, or exacerbating cerebral edema, leading to brain injury [89]. TPGs can improve neurological damage in rats with cerebral hemorrhage, regulate the TH17/Treg balance, reduce brain tissue IL-17 levels, and promote the secretion of IL-10 and transforming growth factor-beta (TGF-β) (Table 2), thus improving symptoms of cerebral hemorrhage [90].

#### 3.5.2. Effects of Treating Acute Cerebral Ischemia and Ischemia/Reperfusion Injury

Ischemic stroke is a common and severe disease with high mortality and disability rates in recent years. Currently, clinical restoration of cerebral blood supply primarily relies on thrombolytic drugs, which are limited by a critical time window and can lead to irreversible secondary damage to brain tissue after reperfusion, known as cerebral ischemia/reperfusion injury (CIRI) [91]. Yang et al. [92] applied TPGs to treat a model of cerebral ischemia/reperfusion induced by incomplete bilateral common carotid artery occlusion. The results showed that TPGs not only improved learning and memory abilities in model mice but also significantly reduced SOD levels, inhibited NO increase, and decreased MDA levels (Table 2). Further studies by Yang et al. [93] found that TPGs extended the gasping time for acute cerebral ischemic mouse models, reduced brain water content, and significantly decreased cerebral capillary permeability. These studies suggest that TPGs have a significant protective effect on both cerebral ischemia/reperfusion and acute cerebral ischemia using mice models. Another study [94] using the aforementioned ischemia/reperfusion model in gerbils observed the effects of TPGs on the brain tissue homogenate brain edema index, SOD activity, MDA concentration, and brain pathology. The results showed that TPGs significantly alleviated brain edema in gerbils, enhanced SOD activity, and reduced MDA concentration (Table 2). Pathological examination revealed that TPGs-treated gerbils had less damage in the hippocampal CA1 region.

In addition, it was found that the ethanol extract of RPR could alleviate brain ischemic damage caused by middle cerebral artery occlusion (MCAO) surgery and reduce oxidative stress damage in neurons induced by H_2_O_2_ [95] (Table 2). It was revealed that the main active components in the ethanol extract of RPR were mostly glycosides, such as paeoniflorin glycoside and paeoniflorin, and their mechanism of action might involve inhibiting ferroptosis and activating autophagy through the PI3K/Akt signaling pathway. Zhao et al. [96] discovered that TPGs were the major active components of RPR natural deep eutectic solvent (NaDES), which significantly reduced the infarct area induced by cerebral ischemia/reperfusion (I/R), lowered MDA content, and increased glutathione (GSH) concentration in MCAO rats. These studies collectively suggest that TPGs have a protective effect on brain neurons, primarily associated with reducing MDA concentration (Table 2).

#### 3.5.3. Effects of Treating Focal Cerebral Ischemia

Xie et al. analyzed the effects of different doses of TPGs on the expression of the Notch intracellular domain (NICD), hairy and enhancer of split 1 (Hes1), and Hes family bHLH transcription factor 5 (Hes5) proteins in rats with a model of focal cerebral ischemia [97]. The results suggested that TPGs reduced the levels of NICD, Hes1, and Hes5 proteins in brain tissue that can be used to treat focal cerebral ischemia. The mechanism may be related to the inhibition of the Notch signaling pathway in brain tissue (Table 2). Ma et al. [98] observed the effects of TPG injection on local cerebral blood flow and behavioral symptoms in rats with focal cerebral ischemia. The results showed that TPGs improved behavioral symptoms in rats with cerebral ischemia, reduced brain edema, decreased the area of cerebral infarction, and alleviated ischemia-induced histopathological changes (Table 2), providing a significant protective effect on focal cerebral ischemia in rats, possibly related to increased blood flow.

**Table 2:** Mechanisms of TPGs in treating cerebrovascular diseases.

Cerebrovascular Disease	Models	Effects	References
Hypertension	SHR	↓ MPV, hs-CRP, sCD40L regulate the balance between serum NO and ET	[88]
	SHR	↓ MMP-9, arterial oxidative stress	[90]
	ICH rats	↓ IL-17, ↑IL-10, TGF-β, regulate the balance between TH17/Treg	[92]
Acute cerebral ischemia and ischemia/reperfusion injury	CIRI mice	↓ SOD, NO, MDA, ↑ learning and memory abilities	[94]
	Decapitated mice, acute cerebral ischemia mouse	↓ brain water, brain capillary permeability, Evans Blue ↑ gasping time	[95]
	CIRI gerbils	↓ MDA, cell damage in CA1 region, ↑ SOD	[96]
	MCAO rats	↓ oxidative stress damage in HT22 cells, MDA, ROS, ferroptosis, ↑ GSH, SOD	[97]
	MCAO rats	↓ MDA, cerebral I/R-induced infarct areas, ↑ GSH	[98]
Focal cerebral ischemia	MCAO rats	↓ NICD, Hes1, Hes5	[99]
	MCAO rats	↓ brain water, cerebral infarct area, ↑ rCBF	[100]
	MCAO rats	↓ MDA, LPO, MMP-9, PERK, CHOP, ATF-6, GRP78, XBP-1↑SOD, CAT, GSH-Px	[101]

(↑ means increase, ↓ means decrease).

In studies involving combination therapy, Gu et al. [99] used TPGs in conjunction with Chuanxiong to treat focal cerebral ischemia. The study found that the optimal ratio of TPGs to total phenolic acids of Chuanxiong (TLPAs) was 7:3. The combination provided more significant protection against focal cerebral ischemia in MCAO rats by alleviating oxidative stress, inflammation, and neuronal apoptosis and protecting the blood–brain barrier (Table 2). Regarding brain energy metabolism, studies found [100,101] that TPGs can improve neurotoxicity induced by strychnos nux-vomica, restore disordered hormone secretion, and enhance brain energy metabolism (Table 2).

### 3.6. Effects of Hepatoprotection

**Figure 4:**
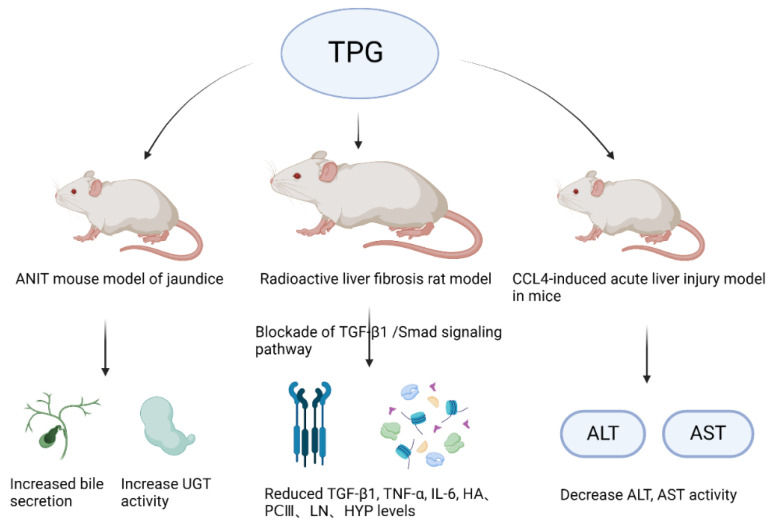
Liver protective effects of TPGs.

The liver, as the largest gland in the human body, is an indispensable organ vital for sustaining life. It performs various physiological functions such as bile secretion, involvement in substance metabolism, excretion and detoxification, phagocytic activity, hematopoiesis, and regeneration. In recent years, with the changes in lifestyle and dietary structure, the incidence of various liver diseases such as fatty liver, hepatitis, liver cirrhosis, and liver cancer has increased significantly year by year [102,103]. Luo et al. [104] studied the action mechanism of TPGs and found that TPGs can reduce the levels of jaundice and enzymes by applying a mouse model of jaundice induced by α-naphthyl isothiocyanate. It was found that TPGs could increase bile secretion in mice, enhance the activity of uridine diphosphate glucuronosyltransferase, promote bilirubin metabolism, increase hepatic enzyme activity, and strengthen the liver’s detoxification capacity. Figure 4 displays the liver protective effects of TPGs.

Liver fibrosis is an inevitable stage in the progression of various chronic liver diseases to cirrhosis, with free radical damage being an important cause. The activation of hepatic stellate cells (HSCs) and the deposition of collagen and the extracellular matrix are critical steps in its development [105,106]. HSCs are the cytological basis for the formation of liver fibrosis, and they are also the main source of the liver’s extracellular matrix (ECM) [107]. Gao et al. [108] found that TPGs had strong in vitro DPPH free radical scavenging and antioxidant activities, and they can inhibit the proliferation of HSC-T6 cells to combat liver fibrosis. It is speculated that their antioxidant activity may be related to their antifibrotic activity. Other studies showed [109,110] that TPGs had a good therapeutic effect on liver fibrosis and liver injury, and it was believed that their mechanism of action may be related to their anti-lipid peroxidation, which is consistent with the aforementioned speculation. Therefore, TPGs may exert antifibrotic action by blocking the TGF-β1/Smad signaling pathway.

Gao et al. [108] studied the hepatoprotective effects of TPGs on a mouse model of acute liver injury induced by CCl_4_ and a mouse model of acute fatty liver induced by DL ethionine. Their research showed that TPGs could reduce the activities of serum alanine aminotransferase (ALT) and aspartate aminotransferase (AST) in the CCl_4_-induced acute liver injury model in mice. They could also decrease the total content of cholesterol (TC) and triglyceride (TG) in liver tissues of the DL ethionine-induced acute fatty liver mouse model, thereby protecting the liver. This also confirmed that TPGs had a regulatory effect on the lipid transport disorder pathway. Moreover, it can be inferred that the mechanism of TPGs on anti-liver injury action may involve promoting the synthesis of apolipoproteins, inhibiting the absorption of exogenous lipids or enhancing the metabolism of hepatic lipids. Ge et al. [111] confirmed that TPGs could reduce serum levels of ALT, AST, alkaline phosphatase (ALP), total bilirubin (TBIL), direct bilirubin (DBIL), and total bile acids (TBAs) in mice with cholestatic liver injury induced by α-naphthyl isothiocyanate, indicating that the hepatoprotective effect of TPGs may be related to the improvement of intrahepatic bile duct epithelial damage, the proliferation of intrahepatic bile ducts, and peri-ductal inflammation caused by bile duct obstruction.

### 3.7. Effects of Antidepressants

Research indicates [112] that increased inflammation can lead to abnormal neuronal changes and induce depressive-like behaviors, with the nucleotide oligomerization domain (NOD)-like receptor complex (NLRP3) being closely associated with inflammation. Su et al. [113] found that TPGs significantly reduced the expression of inflammatory factors and promoted the activation of mitochondrial autophagy, suggesting that TPGs may exert antidepressant and neuroprotective effects by promoting mitochondrial autophagy, thereby inhibiting NLRP3 inflammasome activation and reducing inflammation (Table 3).

In terms of combination therapy, Wu et al. [114] discovered that both TPGs and the total flavones of the epimedium grandiflorum maxim (TFE) had certain antidepressant effects, but TPGs may have a broader impact. The optimal combination ratio of the two was found to be 2:1. The results suggest that the mechanisms of TPGs and the TFE in treating vascular depression may be extensive and possibly related to the monoamine neurotransmitters, such as norepinephrine (NE) and serotonin (5-HT) and their receptors in different brain regions (Table 3).

### 3.8. Anti-Inflammatory Effects

Anti-inflammatory is also one of the modern pharmacological effects studied for TPGs. In the treatment of respiratory systems, TPGs can control the overproduction and secretion of mucin and regulate the protein expression of GSK3β to effectively control inflammation [115]. Xu et al. [116] demonstrated that TPGs could improve lung function in chronic obstructive (Table 3).

Pulmonary disease (COPD) model rats inhibited the NF-κB signaling pathway, increasing forced vital capacity (FVC) and forced expiratory volume in 0.3 s (FEV0.3), reducing lung tissue cell apoptosis rates, and alleviating lung tissue inflammation. Wang et al. [117] showed that TPGs might regulate the NF-κB pathway to increase the levels of anti-inflammatory factors in rats with chronic pelvic inflammation, alleviate uterine tissue inflammation, and improve uterine tissue pathology (Table 3), thereby achieving the therapeutic goal for chronic pelvic inflammatory disease (CPID). Another study [118] indicated that TPGs could exert anti-inflammatory effects by regulating the target protein prostaglandin–endoperoxide synthase 2 (PTGS2) in the metabolic pathways associated with CPID, thereby inhibiting the production of arachidonic acid (AA) (Table 3).

### 3.9. Effects of Other Pharmacologies

Beyond the aforementioned pharmacological effects, TPGs also exhibited immunomodulation, anti-gastric ulcer, etc., effects, as listed in Table 3.

In the realm of immunomodulation, the studies showed that TPGs significantly alleviated clinical symptoms, such as lesion area and the pain index, to treat oral lichen planus (OLP) [119]. Immunohistochemical analysis post-treatment revealed reductions in CD4+ and CD8+ cells, and apoptosis localization markers (Table 3) indicated that TPGs might treat OLP by inducing apoptosis in inflammatory T cells and regulating T cell immunity.

TPGs can exert anti-gastric ulcer effects and improve kidney fibrosis in diabetic nephropathy by enhancing antioxidant capacity. Experimental studies [120] showed that TPGs can strengthen the defense factors and enhance the antioxidant capacity, thereby inhibiting gastric acid secretion and increasing gastric blood flow to achieve anti-gastric ulcer effects (Table 3). Wang et al. [121] induced a diabetic kidney disease (DKD) rat model with intravenous injection of streptozotocin and found that after TPGs treatment, various indexes of the DKD model decreased, including the kidney index, 24 h urinary albumin content, fasting blood glucose (FBG), serum creatinine (Scr), and blood urea nitrogen (BUN), as well as the expression levels of MDA activity, α-SMA, and FN protein in kidney tissue, while the levels of SOD, nuclear Nrf2, and HO-1 protein in the kidney tissue increased. These results suggested that the efficacy of TPGs in improving renal fibrosis in DKD rats was related to the activation of the classic antioxidant stress pathway-Nrf2/HO-1, enhancing the antioxidant capacity in DKD model rats (Table 3).

It was found that the combination of TPGs and total phenolic acid (TPA) of Ligusticum chuanxiong significantly enhanced SOD activity, reduced the levels of MDA, LDH, and NO to alleviate cellular hypoxic damage [122]; it also downregulated the pro-apoptotic gene Bax protein, upregulated the anti-apoptotic factor Bcl-2 protein, significantly increased the Bcl-2/Bax ratio, and inhibited cell apoptosis, thereby protecting vascular endothelial cells. The protective effect against hypoxic damage in human umbilical vein endothelial cells (HUVECs) was optimal when the ratio of TPGs to TPA was 8:2. Diminished ovarian reserve (DOR) is a condition of ovarian dysfunction. Wu et al. [123] found that TPGs can alleviate DOR, with their action mechanism related to activating the follicle-stimulating hormone receptor (FSHR)/cyclic adenosine monophosphate (cAMP)/protein kinase A (PKA)/cAMP response element-binding protein (CREB) signaling pathway to restore ovarian granulosa cell function (Table 3). In addition, TPGs can induce osteoclast differentiation through the NF-κB and mitogen-activated protein kinase pathways and induce resorptive activity in osteoclast differentiation of RAW264.7 cells and PBMCs. The TPGs from the ethanol extract of RPR revealed a certain hypoglycemic effect and can be effective in treating type 2 diabetes [124].

**Table 3:** Mechanisms of action of selected TPG pharmacologic agents (3.7–3.9).

Bioactivities	Models	Pathways	Effects	References
Antidepressant	CUMS mice	-	↓ NLRP3, ASC, pro-caspase-1, caspase-1, IL-1β, GSDMD-N, ↑ PINK, Parkin, ATG5, LC3	[113]
	Depression rats	-	↑ β-adrenergic receptor, 5-HT receptor	[114]
Anti-inflammatory	COPD rats	NF-κB	↓ FEV0.3/FVC, TNF-α, LTB4, TLR4, My D88, p-NF-κB p65/NF-κB p65, ↑ FVC, FEV0.3	[116]
	CPID rats	NF-κB	↓ IL-6, TNF-α, TGF-β1, ICAM-1, MDA, NF-κBp65mRNA, NF-κBp65, ↑ IL-4, IL-10, IL-13, SOD, IκKα mRNA, NF-κBp65	[117]
	CPID rats	AA	↓ PTGS2, WBC, CREA, ALT, ↑ UREA	[118]
Immune modulation	OLP (in vitro)	-	↓ CD4+T, CD8+T, ↑ CD4+/CD8+	[119]
Anti-gastric ulcer	Gastric ulcer mice	-	↓ MDA, gastric fluid, pepsin, ↑ NO, SOD	[120]
Anti-DKD	DKD rats	Nrf2/HO-1	↓ FBG, Scr, BUN, MDA, α-SMA, FN, ↑ SOD, Nrf2, HO-1	[121]
Anti-DOR	DOR mice	FSHR/Camp/PKA/CREB	↓ FSHR, PKAc, *p-CREB*/CREB, E_2_	[123]

(↑ means increase, ↓ means decrease).

## 4. Conclusions and Prospection

In summary, RPR is one of the most commonly used Chinese herbal medicines and is recognized for its clinical effects in clearing heat, cooling blood, promoting blood circulation, and relieving pain. TPGs, as the main active ingredients of RPR, have been found to consist of over fifty monoterpenoid glycosides. Different solvents and extraction methods may affect the content of the extract, but regardless of the extraction method, paeoniflorin content remains the highest. Additionally, paeoniflorin content serves as a crucial indicator distinguishing RPA from RPR, with RPR generally having higher paeoniflorin content due to factors such as processing, origin, variety, and genetics. Apart from paeoniflorin content, other monoterpenoid glycosides, such as oxypaeoniflorin and albiflorin, though present in both RPA and RPR, exhibit significant differences in content. Thus, despite sharing similar main compounds, their pharmacokinetic characteristics may differ.

The pharmacological effects of TPGs mainly manifest in their anticoagulant, antithrombotic, microcirculation improving, anti-cardiovascular, anti-tumor, hepatoprotective, anti-aging, antidepressant, anti-inflammatory, immunomodulatory, etc., properties. Their anticoagulant and microcirculation-improving effects are primarily achieved by prolonging prothrombin and activating partial thromboplastin times, while their antithrombotic effects result from mechanisms such as inhibiting platelet aggregation and reducing blood viscosity, with the inhibition of platelet aggregation possibly mediated by suppressing ADP release. When acting on myocardial cells, TPGs significantly reduce apoptosis and protect against myocardial cell damage by scavenging free radicals and inhibiting oxidative stress reactions, possibly through inhibition of the PI3K/Akt signaling pathway. In contrast to their protective effects on myocardial cells, TPGs induce apoptosis in tumor cells. On one hand, they may not directly exert cytotoxic effects on some tumor cells but can induce apoptosis, proliferation, migration, and invasion by regulating immunity, gene expression, and protein expression. On the other hand, TPGs directly exhibit cytotoxic effects on tumor cells and induce their apoptosis.

TPGs on the brain are primarily evident in their anti-aging and anti-cerebrovascular disease effects. Unlike the mechanisms for anti-aging, the mechanisms for combating cerebrovascular diseases are more diverse, suggesting that TPGs may exert their pharmacological effects against cerebrovascular diseases through multiple pathways and mechanisms.

Hepatoprotection is also one of the significant pharmacological effects of TPGs, with notable therapeutic effects on liver-related diseases such as jaundice, liver fibrosis, acute fatty liver, and acute liver failure. The primary mechanisms of TPGs’ hepatoprotective action may involve promoting the synthesis of apolipoproteins, inhibiting the absorption of exogenous lipids, or facilitating hepatic lipid metabolism. These pharmacological effects are relatively less studied and superficially understood. Moreover, recent research has focused on the combined use of TPGs with other drugs, such as in combination with Coptis chinensis (Huanglian), Ligusticum chuanxiong (Chuanxiong), and Lilium (Baihe).

In recent years, the research on RPR has been deepened in terms of drug source, pharmacological activity, and combination of drugs, but the research on TPGs has been relatively scarce. The 2020 edition of the Chinese Pharmacopoeia only requires the content of monoterpenes, which shows the importance of TPGs to RPR. The study of natural compounds has become a trend, and its future research prospects are also promising. Therefore, it is expected that more in-depth studies on the pharmacological mechanism of TPGs, such as antidepressant, hepatoprotective, hypoglycemic, and other relatively hot topics, will be carried out in order to provide better guidance for clinical use through more in-depth research.

## Figures and Tables

**Table 1:** Main differentiation between RPR and RPA.

	RPR	RPA
Source	Paeonia lactiflora pall. Paeonia veitchii lynch	Paeonia lactiflora pall
Nature and Flavor	Bitter, slightly cold	Acts on the liver and spleen meridians
Meridian	Liver meridian	Liver, spleen meridian
Function	Clears heat, cools the blood, disperses blood stasis, and alleviates pain. Indicated for heat entering the nutritive level, causing rash from warm toxin, vomiting of blood, epistaxis, red and swollen painful eyes, liver qi stagnation causing flank pain, amenorrhea with dysmenorrhea, abdominal pain due to mass formation, injuries from falls and blunt force, and carbuncles and abscesses	Nourishes blood and regulates menstruation, consolidates the yin to stop sweating, soothes the liver to alleviate pain, and calms the liver yang. Indications include blood deficiency manifesting as sallow complexion, irregular menstruation, spontaneous sweating, night sweating, flank pain, abdominal pain, limb cramps, and headaches with dizziness

## Abbreviations

TPGs	Total paeony glycosides	LDH	Lactate dehydrogenase
PLP	*Paeonia lactiflora pall*	ROS	Reactive oxygen species
RPR	*Radix paeoniae rubra*	CTX	Cyclophosphamide
RPA	*Radix Paeoniae Alba*	MDR1	Multidrug resistance1
SD	Sprague Dawley	UC	Urothelial carcinoma
HPLC	High-performance liquid chromatography	SHR	Spontaneously hypertensive rats
NMR	Nuclear magnetic resonance	MRP	Multidrug resistance protein
FAB	Fast atom bombardment	D-gal	D-galactose
MS	Mass spectrograph	MDA	Malondialdehyde
UV	Ultraviolet and visible spectrum	hs-CRP	High-sensitivity C-reactive protein
TLC	Thin layer chromatography	ALT	Alanine aminotransferase
DAD	Diode array detection	SOD	Superoxide dismutase
ESI	Electrospray ionization	FRA	Fructosamine
TOF	Time of flight	TLRs	Toll-like receptors
IR	Infrared spectroscopy	Caf	Caffeine
DVT	Deep vein thrombosis	MPV	Mean platelet volume
GSK3β	Glycogen synthase kinase 3β	HIF-1α	Hypoxia-inducible factor-1α
APTT	Activated partial thromboplastin time	TGF-β	Transforming growth factor-beta
PT	Prothrombin time	NO	Nitric oxide
FIB	Fibrinogen	ET	Endothelin
ADP	Adenosine diphosphate	GSH	Glutathione
AMI	Acute myocardial infarction	NICD	Notch intraCellular domain
Bcl-2	B-cell lymphoma/leukemia-2	Hes1	Hairy and enhancer of split 1
VR	Ventricular remodeling	HSCs	Hepatic stellate cells
HA	Hyaluronidase	ECM	Extracellular matrix
MI	Myocardial infarction	AR	Aldose reductase
tBHP	Ert-butyl hydroperoxide	TC	Cholesterol
I/R	Ischemia/reperfusion	TG	Triglyceride
CK	Creatine kinase	ALP	Alkaline phosphatase
